# Radiation-Induced Salivary Gland Fibrosis: Mechanisms, Emerging Therapies, and Gelatin-Based Bioengineered Models

**DOI:** 10.3390/gels12040296

**Published:** 2026-04-01

**Authors:** Tuan Khang Nguyen, Yazan Mahmoud, Bader Ikbariyeh, Simon D. Tran

**Affiliations:** Faculty of Dental Medicine and Oral Health Sciences, McGill University, 3640 Rue University, Montreal, QC H3A 0C7, Canada; tuan.k.nguyen@mail.mcgill.ca (T.K.N.); yazan.mahmoud@mail.mcgill.ca (Y.M.); bader.ikbariyeh@mail.mcgill.ca (B.I.)

**Keywords:** radiation-induced fibrosis, salivary gland, mesenchymal stem/stromal cells therapy, gelatin-based hydrogels

## Abstract

Radiotherapy is essential for treating head and neck cancer but frequently leads to radiation-induced fibrosis (RIF) in salivary glands (SGs). RIF develops through a cascade of radiation-triggered events, including DNA damage, excessive oxidative stress, and epithelial cell death. Persistent injury can cause cells to become senescent and release inflammatory signals, fueling chronic inflammation. These processes activate pathways, particularly TGF-β/SMAD, resulting in fibroblast activation, myofibroblast differentiation, and extracellular matrix accumulation. Potential treatments include drugs, mesenchymal stem/stromal cell (MSC) therapy, and gene-transfer approaches. In which, MSC therapy is particularly promising as MSCs can migrate to injured tissue and support epithelial regeneration. Yet progress is limited by the difficulty of expanding human acinar cells (ACs) in vitro. To address this gap, tunable alginate–gelatin–hyaluronic acid (AGHA) bioink hydrogels have emerged as a suitable system as gelatin provides adhesion sites for AC attachment and 3D organoid formation, alginate offers tunable mechanical support through ionic crosslinking, and hyaluronic acid contributes essential cues for cell adhesion, migration, and morphogenesis. The aim of this review is to synthesize current understanding of the mechanisms driving RIF, evaluate available therapeutic strategies, and highlight the role of AGHA in generating engineered SG constructs to test MSC therapies for RIF.

## 1. Introduction

Each year, 660,000 head and neck cancer (HNC) cases are newly diagnosed worldwide [1]. Radiotherapy is a key component of treatment for these cancer patients; however, it induces severe damage to salivary glands (SGs) located within the field of ionizing radiation (IR) [2]. Structurally, the SG contains secretory acini composed of acinar cells (ACs) and a hierarchical duct system, supported by a stroma rich in myoepithelial cells, fibroblasts, blood vessels, nerves, and extracellular matrix (ECM) [3]. Picturing SG as a grape tree, the grapes represent ACs whereas the branches are formed by ductal cells. Under normal conditions or during the early stages of moderate stimulation, SG homeostasis is maintained by AC self-duplication or by replacing lost cells through proliferation of acinar progenitor cells [4]. However, prolonged inflammation or severe stimulation can impair this regenerative capacity, resulting in fibrosis and eventual organ failure [4].

IR results in the death of ACs, the cells that primarily secrete saliva [5]. Once ACs are damaged, fibrotic tissue takes over in the stroma, causing radiation-induced fibrosis (RIF) [6]. Clinically, RIF drives chronic SG dysfunction, manifesting as reduced saliva production, xerostomia (dry mouth), dysphagia (impaired swallowing), dental caries, altered taste, oropharyngeal infections, mucositis, and pain [7,8]. Unfortunately, current therapeutic strategies offer little benefit for patients with RIF. Approaches such as artificial saliva products provide only temporary symptom relief [9], and salivary stimulants depend on residual functional glandular cells [10], which are largely absent in individuals with RIF. As a result, these patients suffer considerable morbidity as xerostomia leads to reduced nutritional intake, weight loss, and impaired quality of life [7].

**Figure 1:**
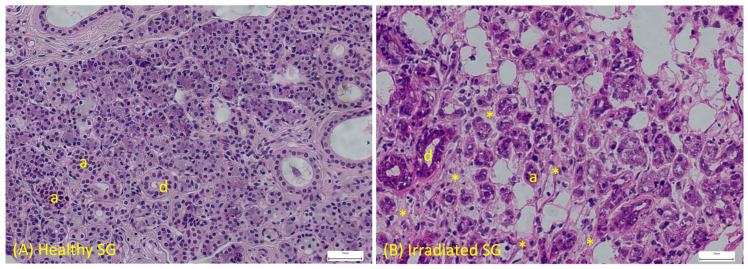
Healthy versus Irradiated human salivary glands (SG). (**A**) H&E staining of human SG with healthy epithelial acini (a) and organized ductal structures (d). (**B**) Irradiated human SG showing atrophic or loss of acini (a), disorganized ductal structures (d), and fibrosis (asterisk). Scale: 50 µm; magnification: 20×.

Fractionated radiotherapy is the most frequent treatment for HNC, typically delivered as 2 gray (Gy) per fraction, five days per week, for a total dose of about 70 Gy [11]. Irradiation of SGs during radiotherapy for HNC causes a significant loss of ACs and stem/progenitor cells [12,13]. As ACs are highly specialized and minimally proliferative, their depletion is compounded by radiation-induced damage to stem/progenitor cells, reducing the gland’s capacity for endogenous repair [13]. The initial parenchymal destruction triggers a series of maladaptive wound-healing responses, including excessive reactive oxygen species (ROS) production, epithelial senescence, apoptosis, and persistent inflammation. These events lead to epithelial to mesenchymal transition (EMT) and activation of profibrotic signaling, particularly transforming growth factor-beta (TGF-β)/SMAD pathway, driving fibroblasts to become activated, promoting their differentiation into myofibroblasts, ultimately resulting in excessive matrix deposition, fibrotic remodeling, and long-term gland dysfunction [5]. During this SG parenchymal destruction, there is a progressive replacement of the lost tissue by ECM proteins and fibrosis (Figure 1).

Fibrosis is an abnormal wound-healing response, marked by excessive tissue repair. When tissue is injured, an essential biological process occurs to replace dead or damaged cells. There are two phases of tissue repair: (1) regeneration, in which damaged cells are replaced by cells of the same type, restoring normal structure and function; and (2) fibrosis, in which connective tissue replaces parenchymal tissue [14]. Although initially beneficial, prolonged activation of tissue repair pathways leads to extensive ECM remodeling, resulting in fibrotic tissue formation [14]. In 5–15% of skin wounds, excessive proliferation of dermal fibroblasts and deposition of ECM may occur, leading to dermal fibrosis such as keloid scarring [15]. For patients receiving radiotherapy, fibrosis often emerges as a major complication due to treatment-induced repetitive injury [16]. RIF of SG is common after radiotherapy for HNC, characterized by replacement of functional parenchyma by components of the ECM. The sustained decline in parenchymal cells after radiotherapy likely results from AC loss that are not replenished by the stem cell population depleted through apoptosis [17]. This remodeling disrupts glandular architecture and impairs secretory function.

Recent advances in SG research have increasingly relied on advanced in vitro models and bioengineered tissue constructs to better replicate the cellular and structural complexity of the native gland. Among these platforms, gelatin-based hydrogels have emerged as particularly promising biomaterials, providing the biochemical cues and 3D microenvironment necessary to support salivary epithelial organization, spheroid formation, and functional regeneration [18]. These innovative systems offer powerful tools for studying radiation-induced injury and for developing next-generation regenerative therapies. This review aims to provide an integrated overview of the mechanisms underlying radiation-induced SG fibrosis, assess available therapeutic interventions, and evaluate the potential of AGHA-based engineered gland constructs for advancing MSC-based regenerative approaches.

## 2. Pathogenesis of Ionizing Radiation-Induced Salivary Gland Fibrosis

**Figure 2:**
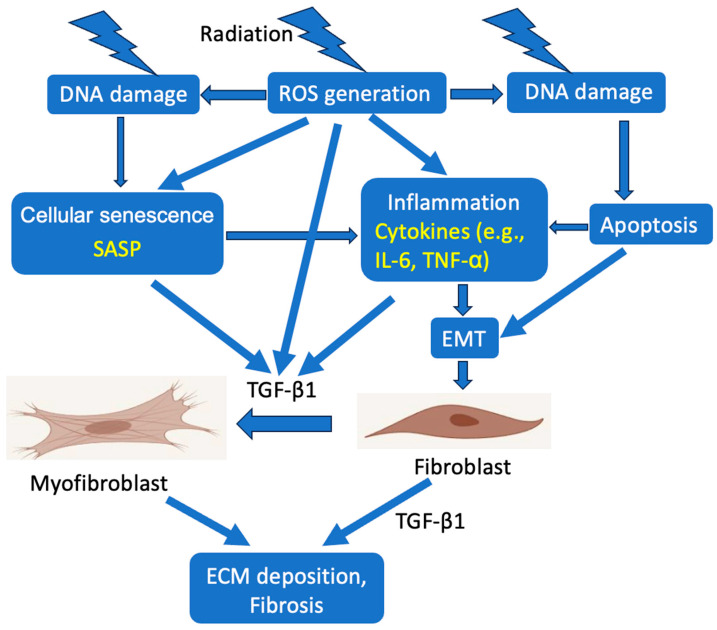
Pathogenesis of ionizing radiation-induced salivary gland fibrosis. Radiation-induced fibrosis develops through DNA damage and excessive Reactive Oxygen Species (ROS) generation, leading to epithelial apoptosis, cellular senescence with senescence-associated secretory phenotype (SASP), chronic inflammation, epithelial-to-mesenchymal transition (EMT), and activation of TGF-β/SMAD signaling, resulting in fibroblast activation, myofibroblast differentiation, and extracellular matrix (ECM) accumulation.

Understanding of the mechanisms of radiation-induced SG fibrosis has largely been derived from studies using animal models. RIF arises from a coordinated sequence of molecular events that begin with acute epithelial injury and evolve into chronic stromal remodeling (Figure 2). In the acute phase following irradiation, immediate DNA damage, rapid ATP release, epithelial apoptosis, and a surge in ROS collectively drive the early damage to SG. Cellular senescence emerges during the transitional period approximately 7 days after radiotherapy and persists into the chronic phase, whereas fibrosis typically occurs at chronic time points, between 4 and 6 months following irradiation [5].

### 2.1. DNA Damage

Immediately after irradiation, a series of signaling pathways are activated that contribute to the development of acute hyposalivation [5]. Studies in mouse SG have shown that DNA double-strand breaks occur within minutes of IR exposure, accompanied by increased γH2AX (phosphorylation of the H2A histone family member X) [19]. Furthermore, DNA repair is compromised as the activity of sirtuin-1 (a stress-induced deacetylase) and the phosphorylation of NBS1 (a DNA repair protein) are reduced [19]. The damage of DNA was also observed in 2D and 3D cultures of SG engineered constructs post-irradiation with 16 Gy [20]. Persistent DNA damage or incomplete DNA repair leads to cell apoptosis, senescence, and toxicity to normal tissues [21,22]. Cellular senescence and apoptosis in response to DNA damage are among the main causes of SG fibrosis.

### 2.2. Reactive Oxygen Species (ROS) Generation

Another acute response to IR that is known to induce severe cellular injury is the generation of ROS [23]. IR was shown to decrease antioxidant enzyme activities and increase oxidative stress marker levels, e.g., xanthine oxidase, in rat SG at day 10 of 5 Gy IR, [24] while mitochondrial ROS levels were increased within 3 days of IR in mouse submandibular glands [25]. Nicotinamide adenine dinucleotide phosphate (NADPH) oxidase, an enzyme responsible for ROS generation, was increased significantly in rat at days 4–7 after IR with 18 Gy [26] and in mice with single 15 Gy irradiation [23]. When produced in excess, ROS disrupts the oxidant-antioxidant equilibrium, leading to oxidative stress that causes cellular damage to biomolecules, such as lipids, proteins, and DNA, and induce cellular senescence or apoptosis [27,28]. In addition, ROS was recently reported to mediate TGF-β1 through several pathways, such as SMAD (suppressor of mothers against decapentaplegic) and MAPK (mitogen-activated protein kinase), which suggests the potential involvement of ROS in glandular fibrosis [29]. In short, ROS contributes to SG fibrosis in two ways: (1) directly, ROS promotes the release of TGF-β1 [30], and (2) indirectly, ROS leads to cellular senescence, which in turn releases a host of inflammatory substances. The roles of TGF-β1, cellular senescence, and inflammation in fibrosis formation will be discussed in detail in the following sections.

### 2.3. Apoptosis

Epithelial apoptosis, occurring within the first few days after irradiation, is considered a primary driver of glandular fibrosis [5,31]. AC apoptosis was observed in several studies in mice between 8 and 72 h after irradiation, peaking at approximately 24 h [5]. This acute response has been quantified using multiple approaches, including via increased mRNA expression of the apoptosis regulators Bax and Puma, caspase-3 cleavage or activity, and TUNEL (terminal deoxynucleotidyl transferase dUTP nick end labelling) assay [5]. Moreover, a study in rats exposed to 18 Gy irradiation showed an increase in TUNEL-positive cells along with enhanced cleavage of caspase-9, the upstream regulator of caspase-3 activation [26]. Another study in irradiated mouse has illustrated that apoptosis in SGs after radiotherapy in the head and neck region is mediated by p53 [12]. Expression of p53 is required for the induction of pro-apoptotic target genes PUMA and Bax, activation of caspase-3, and subsequent apoptosis in SGs, leading to both acute and chronic loss of salivary function; in contrast, p53-deficient mice fail to activate these pathways [12]. Epithelial apoptosis disrupts the integrity of the epithelial barrier, leading to increased barrier permeability and release of pro-fibrotic mediators [32]. While fibroblasts are more resistant to apoptosis, resulting in abnormal fibrotic proliferation. Moreover, epithelial cell death increases the concentration of inflammatory cytokines, e.g., tumor necrosis factor-α (TNF-α), which is involved in regulating the immune microenvironment and tissue fibrosis [32]. Additionally, it contributes to fibrosis formation via the process of EMT [32]. In short, epithelial apoptosis may play a crucial role in the development of fibrosis through a variety of mechanisms.

### 2.4. Cellular Senescence

Senescent cells play an important role in SG inflammation and fibrosis after radiotherapy [20]. Senescence is a cellular state of proliferative arrest that occurs when cells experience significant DNA damage or oxidative stress [28]. Studies in animal SG showed increased markers of cellular senescence and reduced anti-senescence cytokines at 5 weeks post-irradiation, while clinical observations reported similar changes after 4 months [33]. Senescence was indicated by elevated γH2AX and p21 mRNA, appearing by 5 weeks after 20 Gy in minipigs [5]. Additionally, a clinical study found a marked rise in γH2AX-positive senescent ductal cells in submandibular glands resected from patients 4–8 months after irradiation [28]. Senescent cells release a group of bioactive substances, known as senescence-associated secretory phenotype (SASP), including cytokines (e.g., Interleukin-8 (IL-8) and IL-6), chemokines, proteases (e.g., matrix metalloproteinases (MMP) family), and growth factors (e.g., TGF-β and insulin-like growth factor binding proteins (IGFBP)) [34]. In which, TGF-β, a principal regulator of fibrosis, promotes fibroblast proliferation and their differentiation into myofibroblasts to produce matrix components [35]. MMP activity increases after radiotherapy; specifically, MMP2 is consistently upregulated in irradiated SG, observed in porcine parotid tissue and murine submandibular glands [36,37]. MMP-mediated proteolytic activity contributes to matrix breakdown, enabling fibroblast infiltration and remodeling [38]. Cytokines such as IL-8 and IL-6 cause inflammation and affect nearby cells, inducing ECM alterations [34]. Upregulation of Il-6 has been observed in mice SG following radiotherapy [28]. Furthermore, an in vitro study showed that IL-6 enhanced TGF-β expression in SG epithelial cells [6]. The senescence-induced inflammation can promote AC senescence, reduce or destroy existing stem cells that may persist in or migrate to SG tissue preventing natural regenerative processes, and stimulate phenotypic changes in fibroblasts that promote the ECM production, creating fibrosis [20].

### 2.5. Inflammation

Persistent inflammation is a significant cause of SG fibrosis. Although SG has a high regenerative capacity, sustained inflammatory signaling might overwhelm this mechanism and lead to excessive collagen deposition and fibrosis [35]. Immune cells during inflammation, play a crucial role by secreting cytokines and growth factors that sustain fibroblast activation and ECM deposition [20]. Moreover, adenosine triphosphate (ATP) is released from damaged cells leading to inflammation by activating the P2X7 receptor [39]. This process stimulates immune cells to release inflammatory cytokines causing an enhanced immune response, which can lead to cellular apoptosis. Additionally, P2X7 receptor activation has also been found to play a pivotal role in fibrosis, the pathological outcome of most chronic inflammatory diseases [39]. Release of extracellular ATP from parotid cells is a rapid response that occurs immediately after irradiation (2–10 Gy) [40]. In addition, prostaglandin E2 (PGE2), which is produced during inflammation, is significantly elevated in parotid AC cultures 1–3 days after 5 Gy [40]. Hematoxylin and eosin (H&E) staining from human chronic sialadenitis illustrates acinar cell atrophy, ductal cell proliferation, marked inflammatory cell infiltration, and deposition of large amounts of collagen fibers [29].

### 2.6. Epithelial-to-Mesenchymal Transition (EMT)

Sustained inflammation in SGs can lead to the induction of EMT through PI3K/Akt/GSK3β/Snail signaling axis, causing salivary epithelial cell dysfunction and subsequent fibrosis [41]. EMT, characterized by the conversion of polarized epithelial cells into mesenchymal phenotype cells, functions as a supportive pathway for fibrotic remodeling [42]. A group of transcription factors, including Snail Family Transcriptional Repressor Snail/SNAI1, zinc-finger E-box-binding 1 (ZEB1), and Twist, are the master regulators for EMT by repressing the genes encoding epithelial markers and activating mesenchymal marker genes [43]. In this way, damaged epithelial cells can become a supportive contributor for the production of fibroblasts, which are responsible for the pathogenesis of chronic organ fibrosis [41]. A cell undergoing EMT can be identified by a reduced level of epithelial markers (e.g., E-cadherin) and elevated expression of mesenchymal markers (e.g., vimentin) as well as its morphology and behaviors change [42]. In human SG, a study found that the fibrosis in chronic sialadenitis tissue was largely distributed around the atrophied acini as observed by Masson staining [41]. Additionally, colocalization analysis of E-cadherin and vimentin revealed reduced E-cadherin in chronic sialadenitis tissues and newly acquired vimentin in atrophied acini [41]. Moreover, another in vitro study performed by culturing healthy human SG epithelial cells with the IL-6, an important cytokine contributing to acute and/or chronic inflammation, has demonstrated the role of IL-6 in EMT process [6]. It has revealed an increased expression of TGF-β1, vimentin, and collagen type I, together with a decreased expression of E-cadherin in IL-6-treated cells after 24–48 h of culturing. E-Cadherin is a protein responsible for maintaining epithelial cell–cell adhesion. Its loss compromises epithelial integrity and facilitates EMT [6]. Additionally, phase-contrast microscopy showed that epithelial cells underwent marked morphological changes, adopting an elongated phenotype after 72 h of culturing. To summarize, salivary epithelial cells may undergo partial EMT, shifting toward a mesenchymal phenotype in an inflammatory environment, which in part contributes to fibrosis formation [6].

### 2.7. Fibrosis

Tissue fibrosis is the result of a cascade of post-radiation responses. SG fibrosis usually develops 4–6 months after irradiation, though it can appear as early as 8 weeks, and is commonly associated with increased TGF-β levels [33]. TGF-β protein family has been known as a key regulator of fibrogenesis, with TGF-β1 being strongly linked to fibrosis [44]. TGF-β1 increases ROS production and inhibits antioxidant enzymes, leading to redox imbalance; ROS then activates TGF-β1 and mediates TGF-β pathway to form fibrosis, creating a vicious cycle. TGF-β1 acts as a potent pro-fibrotic signal, stimulating fibroblasts, connective tissue, and epithelial cells to synthesize and remodel ECM. Through TGF-β signaling, fibroblasts proliferate, become activated, and differentiate into myofibroblasts, which are the main drivers of fibrogenesis [20,35]. This process is mediated by the TGF-β/SMAD pathway, which activates genes for EMT production and tissue remodeling [44]. Moreover, the signaling axis of TGF-β1/SMAD/Snail was found to regulate EMT-dependent fibrosis [29]. TGF-β has been shown to be elevated in HNC patients and in murine models after radiotherapy [5]. Biopsies of the submandibular gland from advanced oropharyngeal cancer patients treated with fractionated radiotherapy (1.8–2 Gy per fraction, one fraction per day, 5 days per week, total dose 60–70.6 Gy) revealed glandular atrophy, periductal fibrosis, and parenchymal fibrosis [45]. The extent of glandular fibrosis was observed to correlate with the degree of sialadenitis as demonstrated by lymphocytic infiltration [45]. A study in mice exposed to fractionated IR (6 Gy per fraction, 5 fractions) showed severe fibrosis of the submandibular SG (as measured by collagen deposition) after 300 days of IR exposure, and showed upregulation of genes for ECM remodeling and fibrosis (e.g., Col23a1, Mmp2, and Serping1) through RNA-seq analysis [37]. Similarly, minipigs exposed to a single 15 Gy dose of IR revealed extensive glandular fibrosis and upregulation of Serping1 and Mmp2 after 16 weeks of irradiation [37]. An in vitro study concluded that TGF-β1 stimulates human primary SG fibroblasts to produce COL1A1, which was quantified by Western blot analysis [29]. In this study, clinical tissue samples from patients with sialadenitis were also analyzed. The study showed a significant increase in TGF-β1 and the myofibroblast marker α-SMA in the fibrotic tissue of inflamed SG. Their concentrations were significantly correlated with the degree of SG fibrosis [29].

## 3. Therapeutic Strategies

One of the clinical consequences of RIF is xerostomia. Conventional symptomatic treatments, primarily based on saliva substitutes and/or SG stimulation (e.g., chewing gum), have been used to reduce dry mouth symptoms [46,47]. Moreover, pilocarpine and cevimeline, which are parasympathetic nervous system stimulants, have been approved by the Food and Drug Administration (FDA) for the treatment of radiation-induced xerostomia by acting on cholinergic receptors in the SGs [46]. However, treatments based on stimulating SGs to increase saliva secretion are ineffective for fibrotic glandular tissue [9,47]. In contrast, preventive strategies aim to limit acute radiation injury before irreversible fibrosis develops. These include radioprotective agents such as amifostine, HL-003, and N-acetylcysteine amide (NACA), which are administered before or shortly after irradiation to protect tissues from DNA damage, oxidative stress, and early fibro-inflammatory signaling [23,48,49,50]. Finally, regenerative approaches, including gene-transfer therapy and mesenchymal stem cell (MSC) therapy, represent another major therapeutic avenue, aiming to replace or rehabilitate damaged epithelial and progenitor cell populations, offering the potential for meaningful recovery of salivary function [44].

Understanding the pathogenesis of SG RIF highlights TGF-β signaling as a central profibrotic pathway and therefore a main focus of antifibrotic therapeutic research. TGF-β neutralizing antibodies such as Fresolimumab have shown efficacy in reducing cutaneous fibrosis in a phase I clinical trial, although adverse events including dermatologic complications, hemorrhage, and anemia have been reported [51]. Moreover, blockade of TGF-β/SMAD pathway through SMAD-focused inhibitors (e.g., SIS3, Paroxetine) attenuates profibrotic transcriptional responses and improves glandular outcomes in mouse models [52]. Recent work has also identified the ORAI2-mediated calcium signaling pathway as an important regulator of TGF-β1 expression in irradiated SGs; pharmacologic inhibitors of ORAI2 such as SKF96365 and YM58483 effectively reduced glandular fibrosis in irradiated models (Table 1) [53].

Beyond inhibiting the TGF-β pathway, treatment of RIF also targets other key molecular drivers, including cellular senescence, apoptosis, and ROS-mediated damage. Regarding senescence-directed therapies, senolytic drugs, which selectively eliminate senescent cells, offer a promising strategy for reducing SASP-associated inflammatory and fibrotic pathologies [54]. In irradiated mouse models, the senolytic drug ABT263 has been shown to improve SG morphology and function by enhancing stem cell self-renewal and preventing progressive tissue degeneration [55]. Senomorphic therapies are also being explored as an alternative approach; rather than eliminating senescent cells, they act by suppressing harmful SASP signaling, with agents such as rapamycin shown to reduce the expression of multiple SASP-associated cytokines [22,56]. Moreover, several anti-apoptotic strategies have also shown promise. Administration of α-lipoic acid one hour after irradiation significantly reduced apoptotic markers in rats from days 4 to 56 post-IR [26]; and similarly, mice receiving keratinocyte growth factor-1 (KGF-1) one hour before and immediately after 15 Gy irradiation exhibited decreased acinar apoptosis [57]. In addition, MSC therapy has been reported to further diminish apoptosis in irradiated SGs [58,59]. Additionally, treatment with antioxidants, α-lipoic acid, the free radical scavenger Tempo, 3-aminobenzamide, amifostine, NACA, HL-003, and gene transfer therapy has been shown to significantly reduce radiation-induced ROS levels (Table 1) [23,26,49,60,61,62].

Several pharmacologic agents and emerging therapies are outlined below; each designed to prevent or mitigate RIF by directly targeting the molecular pathways that drive fibrotic progression (Table 2).

### 3.1. Amifostine and Melatonin

Amifostine remains the sole FDA-approved medication for preventing xerostomia induced by radiotherapy [46]. A meta-analysis conducted in 2017 showed little evidence of long-term benefit of amifostine for the prevention of dry mouth in patients undergoing head and neck radiation therapy [48]. Furthermore, this drug is often associated with side effects such as hypotension, nausea, and vomiting [48,64]. However, amifostine has been found in several studies for its ability to eliminate excess ROS generated by radiation [62,63], which can lead to fibrosis. Additionally, a study on the submandibular gland of Sprague Dawley rats found anti-inflammatory and anti-fibrosis effects of amifostine and melatonin following radiotherapy [65]. When compared to the radiotherapy group, a significant downregulation in mRNA expression of pro-inflammatory cytokines (TNFα, IL-6) and fibrosis-related factors (TGF-β1, TGF-β2, Col1a1, Col1a2) was observed in the groups of radiotherapy combined with amifostine or melatonin [65].

### 3.2. Metformin

Metformin is widely known for its role in the treatment of diabetes. In 2025, Peng et al. explored the role of metformin in mitigating partial EMT in SG inflammation, a main cause of fibrosis formation [41]. The results showed that metformin reversed EMT in human SG epithelial cells by targeting the PI3K/Akt/GSK3β/Snail signaling axis. Additionally, another study found anti-fibrotic properties of metformin by inhibiting the TGF-β1/SMAD2/3 pathway and interleukin production [29]. In this study, metformin was found to significantly increase adenosine 5′-monophosphate (AMP)-activated protein kinase (AMPK) phosphorylation, contributing in the inhibition of myofibroblast differentiation [78]. Moreover, metformin notably mitigated TGF-β1 stimulation and suppressed the expression of COL1A1 and α-SMA, resulting in decreased collagen production [29]. Furthermore, it significantly reduced IL-1β production, [29] a factor may enhance the role of TGF-β in fibrosis response [79].

### 3.3. Gene Transfer Therapy

The development of biomedical science has been increasingly revealing the enormous potential of gene transfer therapy. It involves applying gene transfer, DNA delivery, and cell transduction techniques to produce high levels of transgenic protein, with the aim of correcting cellular dysfunction or introducing new cellular functions following radiotherapy [80]. A study on murine submandibular glands and porcine parotid gland suggested that CERE-120 (adeno-associated virus serotype 2-based vector encoding human neurturin) could serve as an effective preventive gene therapy to mitigate salivary hypofunction by enhancing gland innervation, restoring immune balance, and reducing fibrosis in SGs [37]. Pre-IR treatment with CERE-120 also reduced the expression of genes related to IR-induced fibrosis and the expression of inflammation-related genes. In another study, adenoviral vectors carrying Shh (Sonic Hedgehog) were delivered into mice submandibular glands 3 days after IR [61]. The study reported a significant reduction in markers of senescence, DNA damage, oxidative stress, and IL-6 by day 90 following IR. Similarly, delivering Shh into minipig parotid glands at 4 weeks after IR was found to increase salivation, enhance parasympathetic innervation, and reduce fibrogenesis in glandular tissue at 20 weeks post-IR [66]. This study also reported an increase in radiation-induced inflammatory cytokines, which was reversed by Shh gene transfer. In short, pre-IR and post-IR gene transfer therapy could be beneficial for mitigating DNA damage, oxidative stress, inflammation, cellular senescence, and fibrosis formation.

### 3.4. Stem Cell Therapy

As mesenchymal stromal/stem cells (MSCs) possess key characteristics including promotion of cell regeneration, antioxidant, and anti-apoptosis, stem cell therapy may be beneficial in numerous SG inflammatory and injury diseases [58,70,71]. Furthermore, MSCs can be mobilized to sites of IR-induced injury, where they contribute to SG repair by transdifferentiating into epithelial cells or releasing paracrine factors [67,68,69]. Several studies have shown that bone marrow MSCs exhibit a protective effect against radiation-induced SG tissue damage [58,72,73]. In these studies, bone marrow MSCs were transplanted into mice 24 h after IR with 15 Gy. When compared to IR-only controls, mice receiving stem cell therapy experienced less SG damage as measured by increased amylase and decreased fibrosis at 3-month post-IR [72,73]. In addition, a reduction in apoptosis was observed after 4 weeks of MSC transplantation [58]. Similarly, numerous studies, using TUNEL assay, have revealed that the number of apoptotic cells is significantly lower in the MSC treatment group compared to control group [74,75,76]. Moreover, treatment with adipose-derived MSCs has been shown to inhibit apoptosis, inflammation, and fibrotic remodeling [59]. A systematic review and meta-analysis in 2024 reported that MSC therapy significantly improved SG function and promoted tissue regeneration after radiotherapy in preclinical in vivo models, with no serious adverse events observed [77]. In this review, multiple included studies revealed improvements in acinar tissue, including increased ACs and more compact acinar structure, along with reduced inflammation, fibrosis, and apoptotic cells following MSC therapy.

However, most of the available evidence to date has been generated from animal models, and translation of these findings into human studies remains in the early investigational stage [52]. A major challenge is the intrinsic heterogeneity of MSC populations, as cells derived from different tissues (bone marrow, adipose tissue, or umbilical cord) display variable immunomodulatory capacity, differentiation potential, and paracrine signaling, leading to inconsistent therapeutic outcomes [81]. Delivery methods present additional limitations: systemic administration often results in inefficient homing to the SGs and significant side-effects, while intraglandular injection offers limited long-term cell retention and carries procedural risks [82,83]. Moreover, key translational barriers remain unresolved, including concerns regarding immune compatibility, potential immune rejection of allogeneic MSCs, and the need to ensure long term safety, particularly regarding unwanted differentiation, tumorigenicity, or chronic immune modulation [83,84]. These challenges underscore the need for standardized MSC characterization, optimized delivery strategies, and the use of controlled in vitro platforms to enhance reproducibility and advance MSC therapy toward clinical application.

#### 3.4.1. Hydrogel-Based Platforms for SG Engineering

To make the application of MSC treatment in humans more feasible, SG engineered 3D constructs recapitulating key features of the native human SG microenvironment should be generated for testing new experimental therapies. Yet, a major challenge is to culture and expand human AC in vitro. To resolve this issue, hydrogel-based platforms for culturing SG cells have been developed. Hydrogels are polymeric networks that retain large amounts of water within their three-dimensional matrix, enabling them to mimic essential ECM-like characteristics. Their biocompatibility and soft, tissue-like mechanical properties further create supportive microenvironments that promote cellular function and tissue stability. Therefore, they are highly suitable for 3D in vitro cell cultures [85]. Hydrogel-based culture of SG cells have achieved remarkable progress, providing effective platforms for cell expansion and supporting the development of artificial SG constructs [86].

Both natural and synthetic hydrogel-based platforms have been used to culture SG cells. Among natural biomaterials, Matrigel, a basement membrane extract derived from Engelbreth–Holm–Swarm mouse tumors, has long been one of the most widely used animal-based biomaterials in SG engineering [87]. Its inherent biocompatibility allows Matrigel to support primary SG cell attachment, survival, and differentiation in 3D culture [86,88]. However, despite these advantages, Matrigel presents several limitations for tissue engineering applications, including poor mechanical properties and significant batch to batch variability [87]. Moreover, as Matrigel is an animal-derived product, it is unsuitable for clinical translation due to immunogenicity concerns and regulatory restrictions [86]. In contrast, polyethylene glycol (PEG) hydrogels, a class of synthetic biomaterials with well-defined composition and high tunability, have also been tested for SG tissue engineering. However, PEG-based systems have thus far been unable to support the reorganization of primary SG single cells into multicellular clusters or well-structured acini [89].

#### 3.4.2. Gelatin-Based Hydrogels

Gelatin-based composite hydrogels, including AG (alginate–gelatin), AGC (alginate–gelatin–collagen), and AGHA (alginate–gelatin–hyaluronic acid), have emerged as promising platforms for SG cell culture. All three formulations exhibit mechanical properties comparable to native SG tissue (~11 kPa) and support 3D spheroid formation. Alginate contributes tunable stiffness through ionic crosslinking, while its combination with natural biomaterials such as gelatin or collagen provides essential cell binding sites necessary for cell–cell and cell–matrix adhesion [86,90]. Gelatin is particularly advantageous due to its biocompatibility, biodegradability, and rapid thermal gelation, and it contains abundant adhesive motifs that promote focal adhesion formation and support salivary epithelial cell attachment, proliferation, and reorganization within the matrix [91]. Hyaluronic acid (HA), a key ECM glycosaminoglycan, further enhances spheroid development by facilitating cell adhesion, migration, and morphogenesis through interactions with acinar cell surface receptors (e.g., CD44) [92,93]. AGHA hydrogels, combining alginate, gelatin, and HA, provide a favorable mechanical and biochemical environment for SG cell expansion, yielding larger, more viable, and structurally organized 3D spheroids compared to other formulations [18]. Together, these findings highlight the promise of MSC-based therapies to treat ionizing radiation-induced SG fibrosis, and underscore the importance of gelatin-based hydrogel systems, such as AGHA, in generating 3D SG spheroids.

## 4. Strengths and Limitations of the Review

A key strength of this review is its integrated approach, bringing together mechanistic insights into radiation-induced SG fibrosis with emerging advances in biomaterials and regenerative therapies. By outlining how oxidative stress, epithelial injury, senescence, and TGF-β/SMAD signaling collectively drive fibrotic remodeling, the review provides a cohesive understanding of disease progression from early cellular damage to chronic fibrosis. Another strength lies in highlighting AGHA hydrogels as a promising platform capable of supporting acinar morphogenesis and enabling more physiologically relevant in vitro models. By linking these engineered systems to the potential of MSC-based therapies, the review identifies a translational pathway for developing therapies that restore SG structure and function.

Despite its comprehensive scope, this review has several limitations. First, as a narrative review, it does not employ a systematic search strategy, which may introduce selection bias and result in omission of relevant studies outside the primary literature streams surveyed. In addition, much of the available evidence for MSC therapy and engineered SG constructs remains preclinical, relying heavily on in vitro systems and animal models that may not fully capture the chronic, multifactorial nature of RIF in humans. The use of AGHA hydrogels in SG research is also relatively new, and long-term data demonstrating their ability to support stable, fully functional acinar phenotypes are still limited. Consequently, while this review highlights several promising therapeutic and bioengineering strategies, it cannot yet fully assess their clinical feasibility, long-term safety, or scalability.

## 5. Conclusions and Future Directions

In conclusion, ionizing radiation triggers a series of interrelated events within SG, beginning with DNA damage and excessive ROS generation that drive apoptosis and cellular senescence. These early injuries subsequently promote chronic inflammation and EMT, which in turn lead to activation of fibroblasts, their differentiation into myofibroblasts through TGF-β signaling, ultimately resulting in excessive ECM deposition and progressive tissue fibrosis. Among the available therapeutic approaches for RIF, MSC therapy has demonstrated the capacity to preserve ACs, reduce apoptosis and inflammation, and mitigate fibrosis. However, the evidence has largely come from studies on animals. To adapt this therapy to humans, the generation of artificial SG constructs is necessary to evaluate the efficacy of the therapy in restoring SG structure and function after IR injury. Additionally, AGHA gels have demonstrated effectiveness in generating 3D SG spheroids with high viability and proliferation, positioning gelatin-based matrices as promising platforms for disease modeling and testing MSC-based interventions. However, additional work is needed to design hydrogel systems that can fully recreate the intricate architecture of the native SG, including not only the highly specialized secretory acinar units but also the surrounding stromal compartment that provides structural, biochemical, and mechanical support. Achieving this level of biomimicry will require the development of advanced biomimetic hydrogel systems with finely tunable mechanical, biochemical, and degradative properties that can guide epithelial morphogenesis, stromal organization, and multicellular communication. To accomplish this, future work must increasingly integrate state-of-the-art bioprinting technologies, which enable spatially controlled deposition of multiple cell types and region-specific bioinks to recreate the hierarchical architecture of the gland.

## Figures and Tables

**Table 1:** Summary of therapeutic strategies targeting radiation-induced SG fibrosis.

Therapeutic Category	Mechanism/Target	Representative Agents
TGF-β Pathway Inhibitors	- Neutralize TGF-β ligand - Block SMAD signaling - Inhibit ORAI2 pathway	- Fresolimumab - SIS3; Paroxetine - SKF96365; YM58483
Senolytics/ Senomorphics	Remove senescent cells/ Suppress SASP factors	- ABT263 (senolytics) - Rapamycin (senomorphics)
Anti-Apoptotic	Prevent radiation-induced apoptosis of acinar cells	α-Lipoic acid; KGF-1; MSC therapy
ROS Scavengers	Reduce oxidative stress and ROS-driven profibrotic signaling	α-Lipoic acid; Tempo; 3-aminobenzamide; Amifostine; NACA, HL-003, Gene transfer therapy

**Table 2:** Summary of Key Therapies for Radiation-Induced Salivary Gland Fibrosis.

Therapy [Related Studies]	Target Mechanism	Adverse Effects/Limitations	Areas Requiring Further Research
Amifostine (FDA-approved) [48,62,63,64,65]	ROS scavenging; anti-inflammatory (↓TNFα, IL-6); anti-fibrotic (↓TGF-β1/2, Col1a1/2)	Hypotension, nausea, vomiting; limited long-term benefit	Need for alternative agents with fewer side effects; optimization of dosing; mechanisms in human SG fibrosis
Metformin [29,41]	Inhibits PI3K/Akt/GSK3β/Snail axis to reverse EMT; anti-inflammatory (↓IL-1β); anti-fibrotic (↓TGF-β1); suppresses TGF-β1/SMAD2/3 signaling→↓COL1A1, α-SMA; ↑AMPK phosphorylation→ ↓myofibroblast differentiation	Possible systemic intolerance in non-diabetic patients; limited SG-specific clinical evidence	Human validation; dose optimization for long-term efficacy with minimal systemic side effects; delivery specifically to SG tissues
Gene transfer therapy (e.g., CERE-120, Shh) [37,66]	Enhances innervation; modulates immune response, reduces fibrosis; decreases senescence, DNA damage & oxidative stress, reduces IL-6	Viral vector risks; immune responses; delivery constraints	Safer vector systems; optimal timing (pre- vs. post-IR); long-term functional outcomes
MSC therapy [58,59,67,68,69,70,71,72,73,74,75,76,77]	Promotion of cell regeneration; anti-apoptotic (↓apoptotic cells); anti-inflammatory; anti-fibrotic	MSC heterogeneity; inconsistent homing; potential immune rejection; tumorigenicity concerns	Standardization of MSC sources; improved delivery methods; engineered scaffolds; human trials

## Data Availability

No new data were created or analyzed in this study.
